# DNA:RNA hybrids form at DNA double-strand breaks in transcriptionally active loci

**DOI:** 10.1038/s41419-020-2464-6

**Published:** 2020-04-24

**Authors:** Aldo S. Bader, Martin Bushell

**Affiliations:** 1 Cancer Research UK Beatson Institute, Glasgow, G61 1BD UK; 2 Institute of Cancer Sciences, University of Glasgow, Glasgow, G61 1QH UK

**Keywords:** Double-strand DNA breaks, Non-coding RNAs, Transcription

## Abstract

The recent discovery of DNA:RNA hybrids, or R-loops, actively forming at DNA double-strand breaks (DSBs) has unlocked fresh insight into how RNA participates in DNA repair. However, the manner of DSB-induced R-loop formation is vital in determining its mechanism of action and is currently under debate. Here, we analyse published DNA:RNA-hybrid sequencing to elucidate the features that determine DSB-induced R-loop formation. We found that pre-existing transcriptional activity was critical for R-loop generation at break sites, suggesting that these RNAs are transcribed prior to break induction. In addition, this appeared to be a specific DSB response at the break, distinct from traditional, co-transcriptionally formed R-loops. We hypothesise that R-loop formation is orchestrated by the damage response at transcriptionally active DSB loci to specifically maintain these genomic regions. Further investigation is required to fully understand how canonical repair processes regulate R-loops at breaks and how they participate in the repair process.

## Introduction

Our genomes are under constant assault by DNA-damaging agents from both exogenous sources, such as UV light, and endogenous sources, such as reactive-oxygen species. These damaging agents can cause the formation of nucleotide adducts, inter-strand cross-links or single-/double-strand breaks (SSBs/DSBs), the repair of which is a major source of mutations within our genome. These mutations have the potential to impair cellular functions, and their accumulation is a significant driving force behind carcinogenesis. DSBs are the most genotoxic form of DNA damage, having the potential to cause a variety of hazardous mutations, including insertions, deletions and even chromosomal translocations^1,2^. In addition, mutations in key DSB repair genes are strongly linked to cancer-prone syndromes, such as ataxia telangiectasia and hereditary breast and ovarian cancer syndrome^3,4^.

DSB repair is performed by the DNA-damage response (DDR), a complex network of pathways that recognises and initiates signalling in response to DSBs. Initial signalling is carried out by the phosphatidylinositol 3-kinase (PI3K)-like kinases (PIKKs), serine–threonine kinases that phosphorylate key signalling proteins, including histone H2AX (to γH2AX), resulting in the recruitment of further downstream factors^5,6^. MDC1 is phosphorylated and recruited to γH2AX, facilitating the recruitment of the E3-ubiquitin ligases RNF8 and RNF168. This initiates a modification cascade resulting in extensive ubiquitylation of the chromatin^7,8^. Ubiquitylation signals recruit 53BP1 and BRCA1 that compete for binding to the break, determining the choice between non-homologous end joining (NHEJ) and homologous recombination (HR), the two major DSB repair pathways^9^. Successful binding of 53BP1 results in NHEJ, where the DNA ends are rapidly processed and ligated, whereas binding of BRCA1 leads to HR, a more complex pathway that utilises the sister chromatid as a template to re-polymerise the broken DNA, thus maintaining repair fidelity^10,11^. Although much faster, NHEJ is considered error-prone as it has no mechanism for checking sequence integrity, and can lead to the aberrant ligation of disparate DSB ends, whereas HR has higher fidelity, but is slower and limited to the S and G2 phases of the cell cycle. The two pathways therefore work concordantly to maintain genome stability and prevent mutations.

Recent publications have uncovered a role for RNA and RNA-binding proteins (RBPs) in DNA repair. A number of publications have shown that RBPs such as Drosha, DICER, Senataxin, THRAP3 and DHX9 are required for successful DSB repair^12–15^. Proteomic investigations into DNA repair have highlighted a broad incorporation of various RBPs, including transcriptional regulation and splicing^14,16,17^, into the DNA-damage response. The exact mechanism by which these proteins function is not fully understood; however, it is becoming clear that they are acting upon RNA molecules at break sites. There is substantial evidence implicating RNA molecules in DNA repair, and several publications have now corroborated the generation of DNA:RNA hybrids (R-loops) at DSBs and importantly demonstrated that these structures are required for efficient DSB repair^18–28^. Interestingly, many RBPs are implicated to function via these DSB-induced R-loops, for example, Drosha has been shown to be required for their generation and RNase H2 and Senataxin for their resolution^19,21,24^. In addition, many core DNA repair proteins have been shown to exhibit strong RNA binding^29^, and some have also shown binding to DNA:RNA hybrids^24,30,31^.

The exact function of these R-loops currently remains uncertain, with the possibilities that they act as damage markers/signal transducers or as templates for high-fidelity DNA repair being amongst the hypotheses proposed. There is also dispute as to the mechanism by which these R-loops are formed, with some publications indicating that they are HR specific, while other reports have stated that they only form at transcriptionally active loci^18,19,24^. The manner in which they are formed is of great importance to the mechanism by which they act, and therefore to fully understand their roles, both the temporal and spatial origin of the RNA species is required. Previous experiments investigating DSB repair have not focused on the factors that determine the formation of DSB-induced R-loops at specific sites of repair. This has led to conflicting reports with opposing models for the function of these R-loops in the repair process. Some suggest that R-loops are produced as a result of de novo transcriptional activity post damage, creating an RNA signal of DNA damage^18,24,32^. Whereas in contrast, others have suggested that the RNA is transcribed prior to break induction and is recruited post damage, acting as a template for high-fidelity repair at actively transcribed regions of the genome^20,33,34^. In addition, it has been suggested that the accumulation of R-loops at DSBs is the result of transcriptional shutdown at DNA breaks^35–38^, leading to RNA-polymerase II pausing and therefore the increased propensity for R-loop formation around these sites^21,39^. These models have very different implications for both the mechanisms and the outcome of DNA repair, and are based on different underlying assumptions regarding the formation of DSB-induced R-loops. Here we will discuss the current data and theories that surround this issue, and re-analyse published datasets to give new insights into the mechanism behind DSB-induced R-loop formation.

### Investigating R-loop coverage

A direct and quantitative approach to analysing R-loops at DSBs is the use of DRIP seq (DNA:RNA hybrid immunoprecipitation followed by high-throughput sequencing) in a cell line with an inducible endonuclease. The Legube lab has pioneered one such cell line, DIvA (damage-induced via ASISI), based on U2OS cells with an ASISI-ER fusion protein that will therefore induce DSBs at ASISI recognition sites across the genome in response to hydroxytamoxifen treatment^40^. They also determined which sites preferentially undergo HR or NHEJ, and profiled the transcriptional activity of each site, providing a powerful resource for this research^41^. These classifications are critical for the analysis of genome-wide data generated in the DIvA cell system as they allow for a much more in-depth understanding of the results. For example, transcriptional activity was quantified via RNA-Polymerase-II S2P ChIP seq providing relative transcriptional activity for each DSB site as a measure of the occupancy of elongating RNA-Polymerase II. Using this alongside the DRIP-seq data allows for the sites to be either grouped or ranked based on their transcriptional activity, and therefore the association of transcriptional activity and DSB-induced R-loop activity can be accurately investigated.

First, we should discuss the analysis of DRIP seq as there are important considerations when representing these data. To successfully understand the data, it is important to use a variety of plots such as boxplots, metagenes and genome browser plots. This variety allows for far more information to be conveyed, giving a broader understanding of the results. In addition, when analysing data from DIvA cells, it is important to compare coverage in a number of ways, for example, comparing repair pathway preference or genomic location of the sites, to further elucidate the mechanisms under investigation. Limiting the visualisation of such data is often done to simplify and reduce its size within figures; however, simplifying data of this complexity often leads to loss of information and in more severe cases skewing and misrepresentation of the results. It is therefore critical that analysts are diligent when interpreting these data to ensure that the results are represented accurately.

### DSB-induced R-loops form in a transcription-dependent manner

**Fig. 1:**
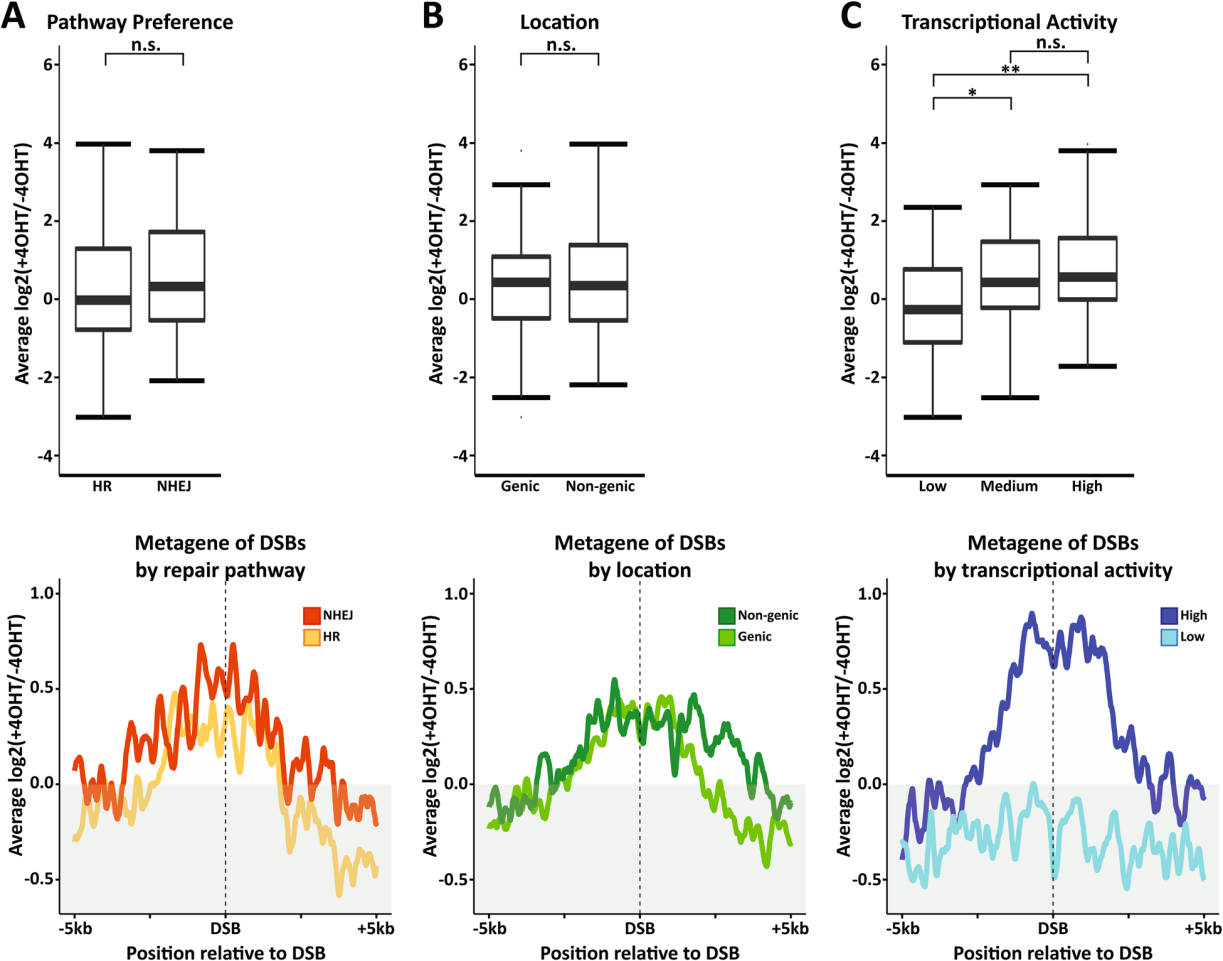
DSB-induced R-loops form in a transcription-dependent manner. Boxplots (top) and metagenes (bottom) of R-loop generation at ASISI-induced double-strand breaks. **a** The 99 breaks are grouped by their repair pathway preference (determined by Aymard et al.^41^). **b** Breaks are grouped by their genomic location status. **c** Breaks are grouped by their relative transcriptional activity (determined by Aymard et al.^41^). DRIP read coverage was calculated and normalised to the total read number; then the log2 fold change of broken (+4OHT) over unbroken (−4OHT) was calculated. Statistics were calculated using a directional, unpaired Wilcoxon signed-rank non-parametric test, *p < 0.05, **p < 0.01.

To gain a greater mechanistic understanding of DSB-induced R-loop formation, we re-analysed the data published by the Legube lab^21^ using a variety of analytical approaches and visualisation techniques. When comparing DSB-induced R-loop formation at HR-repaired sites to NHEJ-repaired sites, there is no significant difference between the two groups, suggesting that R-loop formation at DSBs is independent of downstream repair pathways (Fig. 1a). This has been corroborated by previous DRIP-seq analysis^19^. In addition, by grouping the sites into intragenic and intergenic, we can again see that this has no impact on DSB-induced R-loop formation, suggesting that genomic location is also not a determining factor (Fig. 1b). This has been corroborated previously using the same data set^24^. In this previous publication, this was used as evidence of the R-loops forming independent of transcriptional activity of the locus; however, genic versus non-genic does not denote transcriptional activity, and in actuality ASISI recognition sites are commonly found in promoter regions that have high transcriptional activity despite being classed as non-genic^42,43^. To address this, we grouped the sites into high and low transcriptional activity. This showed that there is a significant increase in DSB-induced R-loop generation at sites with high transcriptional activity over those with low activity, indicating that the pre-existence of transcription is a significant contributor to the formation of these R-loops (Fig. 1c). A dependence on transcriptional activity was also found by previous DRIP-seq analysis and has been suggested by a number of publications investigating RNA-dependent DNA repair (RDDR)^18–22,34,44^. This is important as it indicates that the RNA is produced prior to induction of the break and is recruited in response to damage. Some publications have also claimed that this is evidence of RNA-templated DNA repair, as an RNA molecule transcribed prior to break induction can theoretically be used as a source of genetic information to prevent mutation, similar to the process of HR^20,34,44^.

From this analysis, it would appear that transcriptional activity is a decisive factor in the generation of R-loops at DSBs, whereas downstream repair pathway and break location are dispensable. One explanation for the formation of R-loops independent of downstream repair pathway is the presence of a separate pathway entirely that is centred around RNA^45,46^. The link between DSB-induced R-loops and transcriptional activity could also be a key feature of canonical DSB repair, which would explain the preferential repair of transcribed loci that has been observed in multiple studies^41,47^.

### Transcriptional activity is a central driving force behind DSB-induced R-loop formation

**Fig. 2:**
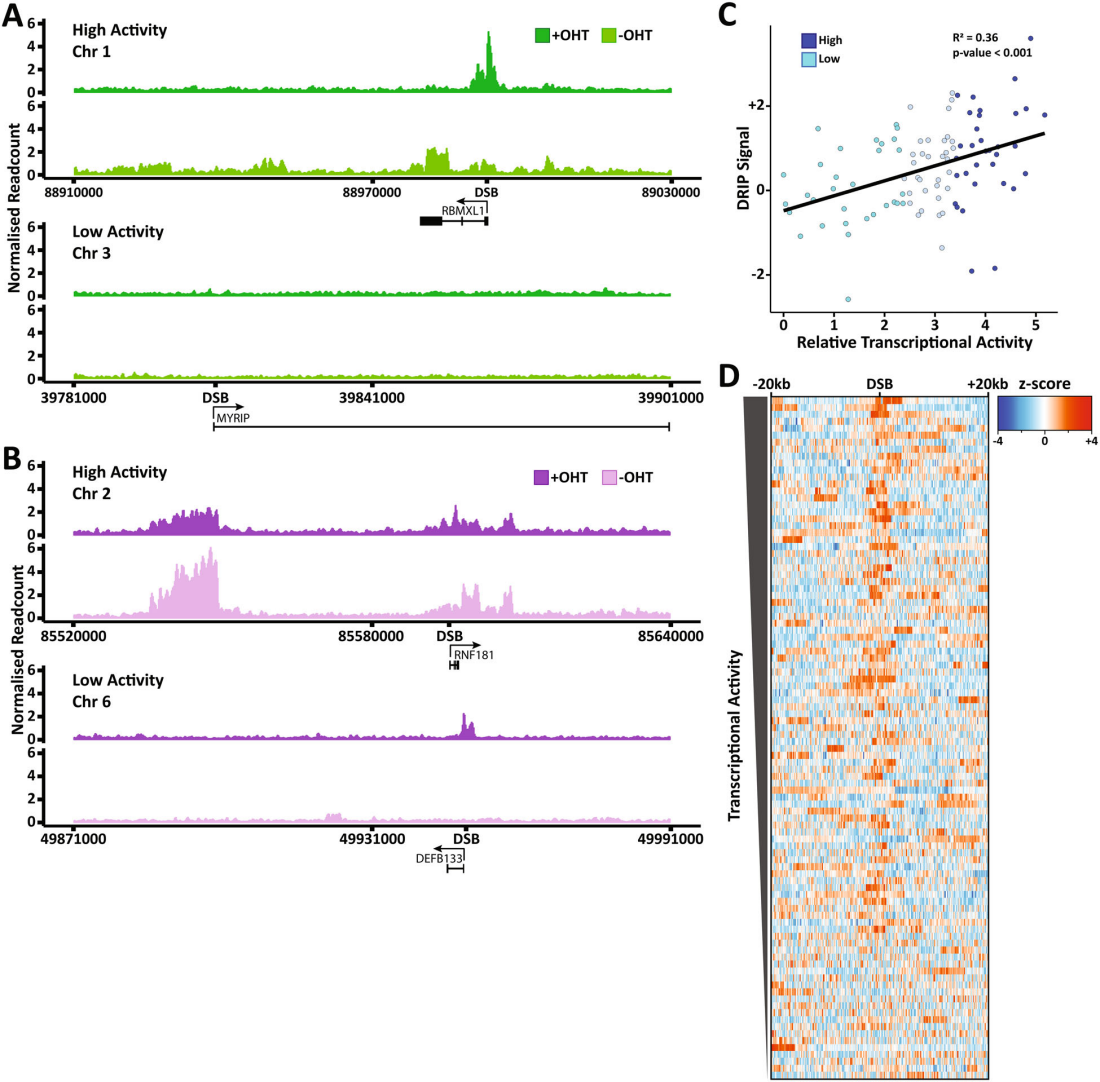
Transcriptional activity is a central driving force behind DSB-induced R-loop formation. **a** Genome browser plots of ASISI-induced double-strand breaks with and without break induction where R-loop generation correlates with transcriptional activity. Two individual sites are shown; one with high transcriptional activity (top) and one with low transcriptional activity (bottom). **b** Same as **a**, except at sites where R-loop generation does not correlate with transcriptional activity. **c** Correlation plot of relative transcriptional activity against the break-induced R-loop generation at each of the ASISI-induced double-strand break sites. Correlation and *p* values determined by Pearson correlation testing. **d** Heatmap of break-induced R-loop generation around each ASISI-induced double-strand break site with the sites ordered from the highest (top) to the lowest (bottom) transcriptional activity.

To gain a greater understanding of the mechanism behind DSB-induced R-loop generation, we conducted a more in-depth analysis of the DRIP-seq data and found that looking at individual break sites sheds more light on this response. Looking at a single high transcriptional activity site shows that in comparison with the untreated control, there is a complete loss of R-loops in the region spanning up to 100 kb from the break, but at the break itself, there is a sharp and specific gain of R-loops (Fig. 2a). Since canonical R-loops are formed behind transcription bubbles, the loss of R-loops in response to DSBs is consistent with reports of transcriptional shutdown at breaks^35–38^. The enrichment seen in close proximity to the break greatly exceeds that of the baseline, suggesting that these R-loops are formed independently of canonical R-loops upon break induction instead of R-loops being retained in proximity to the site. Together, this implies that transcription is shut down around DSBs, resulting in the loss of R-loops, but that the damage response then results in the formation of new R-loops around formerly transcriptionally active break sites. Despite this response being clearly visible at many break sites, there are also some that do not fit this trend. While the number of these sites is low, some sites with high transcriptional activity show no visible enrichment of R-loops, despite still seeing the loss of canonical R-loops, and there is also a minority of sites with low transcriptional activity that show a small enrichment at their breaks (Fig. 2b). Though these sites are low in activity, it is worth noting that they still show RNA-polymerase II occupancy^21^, and therefore this does not necessarily go against the model, but instead suggests that DSB-induced R-loops likely have other determinants that contribute to their formation, similar to the complex regulation of canonical R-loop structures^48–51^.

We next employed correlation analysis to study the association between transcriptional activity and DSB-induced R-loop generation at every site on a continuous scale. Plotting R-loop induction against relative transcriptional activity for each break site shows a positive relationship between the two (Fig. 2c). In addition, statistical testing using Pearson correlation found that there is a highly significant positive correlation between them (Fig. 2c). This indicates a direct relationship between R-loop induction at DSBs and transcriptional activity. However, although the trend is clear, there are a number of outliers. To further investigate this correlation, we therefore used a heatmap to show the distribution of R-loops across each of the 99 DSBs analysed, ordering the sites by descending transcriptional activity (Fig. 2d). This confirmed the previous result, showing a clear trend between R-loop induction and transcriptional activity with some variation. Although this supports the hypothesis of DSB-induced R-loops being transcription dependent, it again highlights that other significant factors are at play. The complexity of DSB repair and the number of elements that can influence its efficiency and outcome mean that further research is needed to fully understand this mechanism.

## Discussion

It is clear that R-loop formation at DSBs is dependent on the transcriptional activity of the locus. Furthermore, this association appears to be a direct relationship, indicating that transcriptional activity drives DSB-induced R-loop formation via the availability of pre-existing transcripts at DSBs. This supports the models of either the DDR orchestrating this R-loop formation to facilitate repair, or their formation being the result of transcriptional pausing in response to damage. However, there are clearly other determinants that may not be discernible by DRIP-seq analysis.

The association of RNA and R-loops with DNA repair goes far beyond these sequencing results. There is also evidence of DNA repair factors utilising RNA in repair processes. Recent publications have shown that the DNA repair protein RAD52 can anneal RNA to double-stranded DNA via inverse-strand exchange to create an R-loop^31^. RAD52 was also found to facilitate DNA repair in neuronal cells in an R-loop-dependent manner^20^. Furthermore, the NHEJ complex was found to associate with pre-mRNA in the form of an R-loop and to utilise it for error-free repair, but only at transcriptionally active loci^44^. This again highlights the association of R-loops with both HR and NHEJ processes, and suggests that the RNA is either involved in both pathways, or is a distinct pathway that uses components from both HR and NHEJ to facilitate enzymatic processes^45^.

It is still unclear how canonical DSB repair processes are regulated, and what determines repair efficiency at different genomic loci. The nature of the DSB is thought to be a key determinant of the choice between NHEJ and HR. Simple break ends are considered to be preferentially repaired by NHEJ, whereas more complex breaks, such as those with long overhangs, are thought to be preferentially repaired by HR^52,53^. How these features affect RDDR is still to be investigated and is beyond the scope of the analysis shown here as the DIvA cell system, although extremely versatile, consistently produces the same type of DSBs with short overhangs.

The data here strongly support that DSB-induced R-loops are formed from pre-transcribed RNA. How this interacts with canonical processes to facilitate DNA repair remains unclear. The examination of how features such as sequence repetition, chromatin conformation and DSB nature influence the dynamics of RDDR will likely yield significant insights into how the mechanism as a whole functions. As our understanding of this mechanism progresses, our ability to query and investigate these in-depth questions will continue to grow, and we hope that our results here contribute to developments that shed further light on these complex processes.

## Materials and methods

### DRIP-seq raw-data processing

The DRIP-seq data analysed were previously published by Cohen et al.^21^, and were downloaded from Array Express using accession number E-MTAB-6318. Fastq files were trimmed using Cutadapt to remove reads under 5 nt in length and to trim ends with Q scores below 20. The filtered reads were then aligned to the human genome version GRCh38 using Bowtie2 with default settings, outputting in SAM format. SAMs were then sorted into BAM format and indexed using Samtools Sort and Index, respectively. Read coverage was calculated across 20-kb bins around the ASISI recognition sites using Samtools Depth to give single-nucleotide resolution read coverage. A custom Awk script was then used to normalise the read coverage to the total number of aligned reads by dividing the coverage at each nucleotide by the total number of aligned reads for that condition, which was determined using Samtools View. A further custom Awk script was then used to determine the log2 fold change of +OHT/−OHT- normalised read coverages of each nucleotide. This resulted in a file containing three columns: chromosome, coordinate and normalised log2 fold change coverage, which was then used downstream for plot generation.

### Metagene and heatmap generation

A custom R script was used to load the coverage files into R, and based on the chromosome and coordinates, convert the coordinates to a position relative to the ASISI cut site and append metadata such as transcriptional activity, repair pathway preference and genic status. Metagenes were plotted by first smoothing data using a 200-nt moving average window, which was then used to create plots via ggplot2 using “geom_line” and colouring based on the various metadata attributes. Heatmaps were plotted using the “heatmap.2” function of gplots. The data set was ordered based on descending transcriptional activity before the heatmap was plotted, and therefore no clustering or dendrogram formation was used.

### Boxplot and correlation plotting

The coverage file was imported and processed as with the metagene/heatmaps. These data were used to generate a singular coverage peak for each site by taking the mean of the log2 fold change of a 1-kb region centred on the break (−500nt − +500nt). Boxplots were then plotted using “geom_boxplot” and correlation plots were plotted using “geom_point” and overlaying a linear regression line with “geom_smooth”. Boxplot statistical analysis was completed using an unpaired, directional Wilcoxon rank-sum test with Bonferroni correction.

### Genome browser plot generation

For browser plots, read coverage was recalculated across a 200-kb window around selected ASISI cut sites. This was processed in the exact same way as all other plots, except that no log2 fold change was calculated; instead, files of the normalised read coverage for –OHT and +OHT were imported separately. These were loaded into R the same as for previous plots, but the data were then smoothed using a 500-nt moving average window and then binned into 200-nt bins to reduce processing load. Ggplot2 was then used to create the plots via “geom_bar”. The gene tracks were obtained using the UCSC Genome Browser and overlaid in Adobe Illustrator.

### Software versions

Cutadapt—1.18

Bowtie2—2.2.5

Samtools—1.7

R—3.4.3

ggplot2—3.2.0

gplots—3.0.1.1

## Data Availability

All scripts used in this analysis, including a shell script containing all raw-data-processing steps, have been uploaded to the GitHub repository https://github.com/R11-Beatson/drip_seq.
